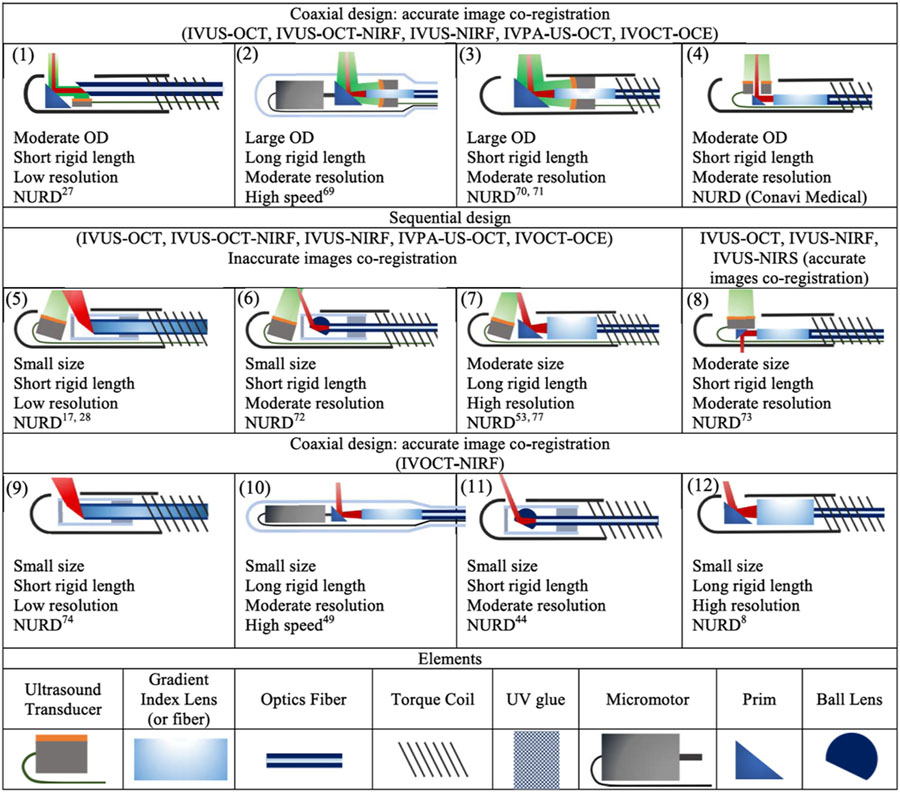# Multimodal intravascular imaging technology for characterization of atherosclerosis

**DOI:** 10.1142/s1793545820300013

**Published:** 2020-01

**Authors:** Yan Li, Jason Chen, Zhongping Chen

**Affiliations:** 1 Beckman Laser Institute, University of California, Irvine 1002 Health Sciences Road, Irvine, CA 92617 USA; 2 Department of Biomedical Engineering University of California, Irvine, CA 92697-2700 USA

**Keywords:** Multimodal, intravascular imaging, photoacoustic, ultrasound, optical coherence tomography, near-infrared fluorescence, spectroscopy, atherosclerosis, imaging probe

## Abstract

Early detection of vulnerable plaques is the critical step in the prevention of acute coronary events. Morphology, composition, and mechanical property of a coronary artery have been demonstrated to be the key characteristics for the identification of vulnerable plaques. Several intravascular multimodal imaging technologies providing co-registered simultaneous images have been developed and applied in clinical studies to improve the characterization of atherosclerosis. In this paper, the authors review the present system and probe designs of representative intravascular multimodal techniques. In addition, the scientific innovations, potential limitations, and future directions of these technologies are also discussed.

## 1. Background

Cardiovascular disease is the leading cause of death in developed countries, and ruptured atherosclerotic plaques are the main cause of acute coronary events.^1–5^ Early detection and characterization of vulnerable plaques are the first and necessary step in preventing lethal consequences and for selecting proper interventional techniques. Thin fibrous cap, large lipid pool, and inflammatory reaction have been demonstrated as the criteria to estimate the presence of vulnerable plaques. Clinically, angiography is the routine practice to detect atherosclerotic plaques by visualizing the coronary arteries in two-dimensional (2D) space. While stenosis can be identified using this method, tissue level information of the arterial wall is lacking. To address this issue, various intravascular imaging technologies — including optical coherence tomography (OCT), ultrasound (US), near-infrared fluorescence (NIRF), photoacoustic (PA), and optical coherence elastography (OCE) — have been developed to characterize the thin fibrous cap, lipid pool, localized inflammation, and biomechanical properties related to atherosclerotic plaques by quantifying the layered architecture, compositional data, and mechanical properties of coronary arteries.^2,6–17,75^

Intravascular ultrasound (IVUS) is the most commonly used clinical imaging technique for plaque diagnostics.^18,19^ IVUS can provide cross-sectional structural images of the coronary vessel with a resolution of ~ 100 *μ*m and an imaging depth of ~ 7 mm. In routine practices, IVUS is used to visualize lumen geometry and layered architecture of the arterial wall as well as to classify the plaque.^20^ The characterization of plaque lesions relies on tissue morphology and pixel intensity and, therefore, has a limited accuracy for studying plaque composition. Moreover, the resolution provided by IVUS cannot visualize micro-features of the plaque that are associated with increased vulnerability, such as neo-vessels, macrophages accumulation, and thin fibrous caps.

As the light analogy to IVUS, intravascular optical coherence tomography (IVOCT) is an imaging technique that is based on low coherence interferometry to provide cross-sectional images of the arterial wall with a superior resolution of 1–15 *μ*m.^21,22^ Benefiting from the micron-scale resolution, neo-vessels, micro-calcifications, thin fibrous caps, and microphage accumulations can be visualized. IVOCT can also classify the plaque lesion based on the analysis of microscopic structure and image intensity.^20^ However, similar to IVUS, these indirect measurements have limited sensitivity for identifying plaque composition. Furthermore, full-depth visualization of the lipid pool is often unachievable due to its limited penetration depth.

Intravascular near-infrared fluorescence or spectroscopy (NIRF or NIRS) is capable of providing molecular contrast with high sensitivity.^20,23–26^ In NIRF, a contrast agent (e.g., indocyanine green or ICG) is administrated to bind to lipoproteins and accumulate at the sites of the inflamed tissues, allowing the detection of lipid content and inflammatory reaction. Recently, differentiation of plaque types has been demonstrated via autofluorescence with a 633 nm excitation wavelength. In addition, NIRS is able to distinguish the compositions of plaque based on the unique absorption spectrum of each substance without the need for contrast agents. Although molecule contrast can be obtained by NIRF (or NIRS), the related depth information is lacking, hence the limited capability of plaque characterization.

Intravascular photoacoustic (IVPA) utilizes a nanosecond laser to excite biological tissues for the generation of photoacoustic signals caused by localized thermal expansion.^17,27,28^ IVPA is based on tissue absorption contrast and has the ability to visualize depth-resolved composition of atherosclerotic plaque. Additionally, multiple wavelengths of laser pulse excitation can be applied to differentiate different compositions of the arterial wall. IVPA can also be seamlessly integrated with IVUS as it uses a US transducer to detect acoustic signals.

Intravascular optical coherence elastography (IVOCE) is a functional extension of IVOCT, and it allows the mapping of arterial wall elasticity by measuring the localized tissue displacement induced by an excitation force.^16,29–31^ As IVOCE relies on IVOCT that has a micron-scale resolution and subnanometer displacement sensitivity, it has the advantage of high sensitivity for quantifying arterial wall mechanical properties. Characterization and quantification of plaque lesions using IVOCE have been demonstrated recently.^16^

Each imaging technique has unique features as well as limitations, which are summarized in Table 1. Presently, since no single technique can provide a complete assessment of the plaque, several imaging methods are often performed in sequence to achieve a comprehensive evaluation.^32^ While the sequential imaging approach can compensate for limitations of each individual technique, the increased X-ray exposure, procedure length, and associated risks cannot be overlooked. As multiple imaging probes are required, repeated probe insertions to the arteries are required, and the associated costs (e.g., guide wires, sterilization, etc.) also increase significantly. In addition, since data acquisition is performed individually, image co-registration is necessary, which is often performed off-line manually or semi-automatically. Not only is image co-registration a tedious and time-consuming task, it also has limited accuracy due to human error and interobserver variances. Therefore, a technique that can simultaneously perform multiple imaging technologies through a single intravascular imaging probe would greatly improve clinical outcomes in cardiology.^33,34^ In this paper, we review the current designs of representative intravascular multimodal imaging modalities, including the respective systems and probes. *In vivo* and *ex vivo* studies on plaque characterization are also presented. Lastly, potential improvements and future directions are discussed.

## 2. System Design

### (1) IVUS-OCT

**Fig. 1.**
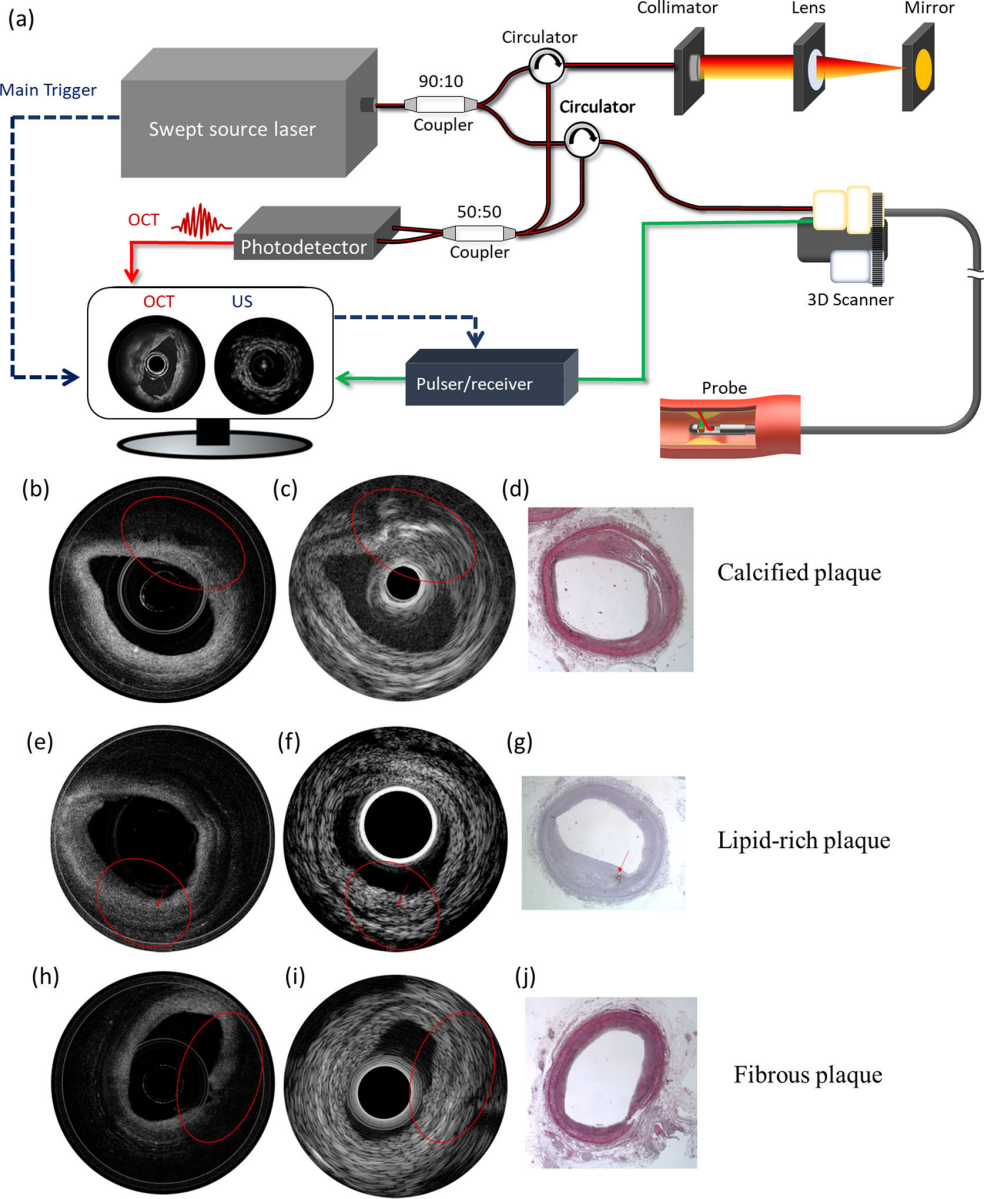
Integrated IVUS-OCT. (a) A typical IVUS-OCT system schematic. (b) OCT, (c) IVUS, and (d) histology of a calcified plaque. (e) OCT, (f) IVUS, and (g) histology of a lipid rich plaque. (h) OCT, (i) IVUS, and (j) histology of a fibrous plaque. Adapted from Refs. 39 and 40.

An imaging system integrating IVUS and IVOCT (IVUS-OCT) is capable of visualizing the morphology of coronary arteries with high resolution and large penetration depth. The first design of an IVUS-OCT system was demonstrated in 2010.^35–39^ Currently, several companies are in the process of commercializing IVUS-OCT technology, including Conavi Medical, Inc. and OCT Medical Imaging, Inc. Figure 1(a) depicts a typical IVUS-OCT system design. The trigger signal from the swept-source laser is used to synchronize the short acoustic pulse emission as well as the data acquisition of IVUS and IVOCT. For proximal scanning, a three-dimensional (3D) scanner consisting of an optical rotary joint, electrical slip ring, rotary motor, and translation stage is used for performing volumetric scanning. Figures 1(b)–1(j) show the representative IVUS-OCT images and histology from a rabbit experiment.^40^ Figures 1(b)–1(d) represent OCT, IVUS, and histology of a calcified plaque, respectively. Due to the limited penetration depth, the calcification cannot be readily observed via OCT but is clearly demonstrated in the IVUS image. Figures 1(e)–1(g) and Figs. 1(h)–1(j) show OCT and IVUS images of a lipid-rich plaque and fibrous plaque, respectively, as well as the corresponding histology. The OCT images reveal the structural similarity between the lipid-rich and the fibrous plaques [Figs. 1(e) and 1(h), respectively], and the co-registered IVUS images help further characterize the pathology [Figs. 1(f) and 1(i), respectively], demonstrating the improved capability of integrated IVUS-OCT in identifying and characterizing plaques.

Although visualization of plaque structural information on different spatial scales enabled by IVUS-OCT can improve atherosclerosis characterization, several limitations still remain. Firstly, the highest IVUS-OCT imaging speed reported to date is 72 frames per second (fps).^39^ A higher imaging speed can decrease the dosage of flushing agent and therefore reduce the associated risks.^41^ Currently, the imaging speed is mainly limited by IVUS due to the slow operation speed of the electrical slip ring which is less than 100 revolutions per second. A mercury slip ring that operates at a much higher speed can be introduced to address this limitation. In addition, a micromotor-based distal scanning method could be an alternative solution. Nevertheless, US propagation speed will always be the limiting factor. Secondly, current IVOCT systems commonly utilize a swept-source laser with a center wavelength of 1.3 *μ*m, which has limited penetration depth. It is possible to use a swept-source laser with a longer center wavelength, such as 1.7 *μ*m, to enhance the penetration depth while maintaining the ability to visualize the microstructure of the arterial wall.^8^ Lastly, a radiofrequency backscatter spectrum analysis can be incorporated into the post-processing procedure of the US data to provide quantification of plaque composition.^42,43^

### (2) IVOCT-NIRF and IVOCT-NIRS

**Fig. 2.**
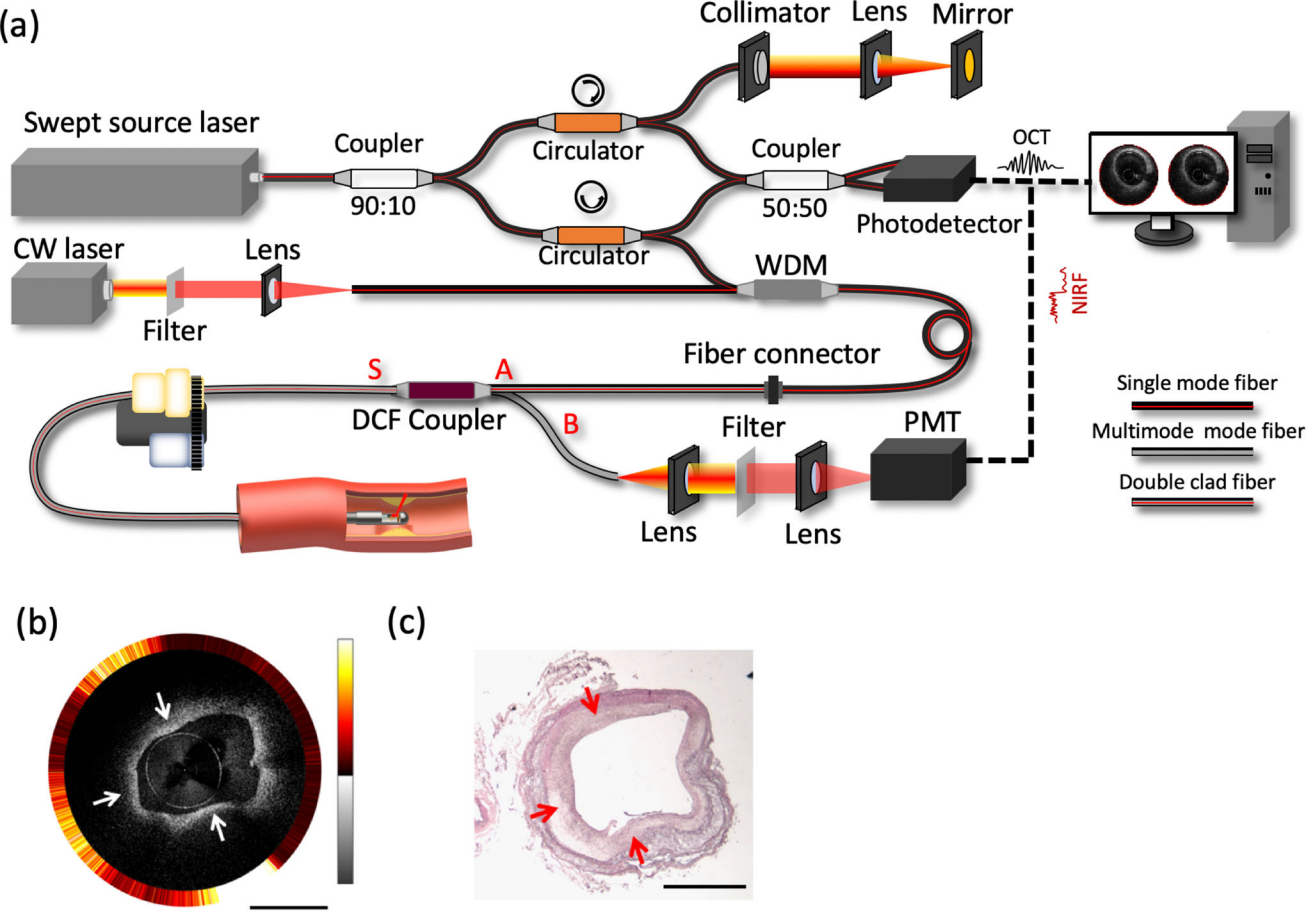
Integrated IVOCT-NIRF. (a) A typical IVOCT-NIRF system schematic. WDM: wavelength-division multiplexing. DCF: double-clad fiber. PMT: photomultiplier tube. CW: continuous wavelength. (b) Representative IVOCT (inner) and NIRF (outer) image of a rabbit aorta in which high NIRF signals, denoted by the white arrows, are observed, indicative of macrophage aggregation. In the OCT image, thickening intimal is noted, which is supported by the histological reading. (c) The corresponding histology of (b). Scale bars: 1 mm.

To provide the molecular contrast while maintaining the ability to visualize the layered architecture of the arterial wall, IVOCT-NIRF systems have been developed, including ICG-based NIRF and autofluorescence NIRF.^44–47^ An exemplary system setup is shown in Fig. 2.^48^ The trigger signal from a swept-source laser is used to synchronize the data acquisition of IVOCT and NIRF signals. A wavelength-division multiplexer (WDM) is utilized to combine the OCT light and NIRF light into a single optical fiber. A double-clad fiber (DCF) coupler at the distal end transmits the combined light beam, after which the back-scattered OCT light and NIRF emission light from the sample are collected and propagated back to the coupler. The OCT light returns through the same path, generates interference patterns with the back-reflected light from the reference arm, and then is detected using a photo-detector. The NIRF emission light separates from the OCT light by propagating through the DCF coupler and then is detected by a photomultiplier tube. A free space optical path can also be used for signal transmission and collection in place of a DCF coupler. For radial scanning, either distal or proximal scanning can be applied. The imaging speeds are up to ~ 200 fps and 5600 fps for proximal scanning and distal scanning, respectively.^49,76^ The first clinical *in vivo* imaging of human coronary arteries using an IVOCT-NIRF multimodal system was reported in 2016.^46^ In this study, IVOCT-NIRF images from 12 patients with coronary artery disease were obtained to demonstrate the capability of this technology to characterize plaques. In addition, IVOCT-NIRS has also been developed to explore the composition of the coronary artery.^45^ Based on the unique absorption spectrum of biological tissues, the plaque composition can be identified using IVOCT-NIRS.

At the current state, the feasibilities and sensitivities of IVOCT-NIRF and IVOCT-NIRS still require further evaluation. Since the NIRF signal is sensitive to distance, the NIRF intensity calibration algorithm can be further improved to provide accurate quantitative analysis of lipid components. IVOCT-NIRS has a high specificity for different plaque types, but the adequacy of the penetration depth to cover the plaque is still unclear. In addition, both NIRF and NIRS lack depth information and cannot provide tomographic mapping of tissue composition.

### (3) IVOCT-OCE

**Fig. 3.**
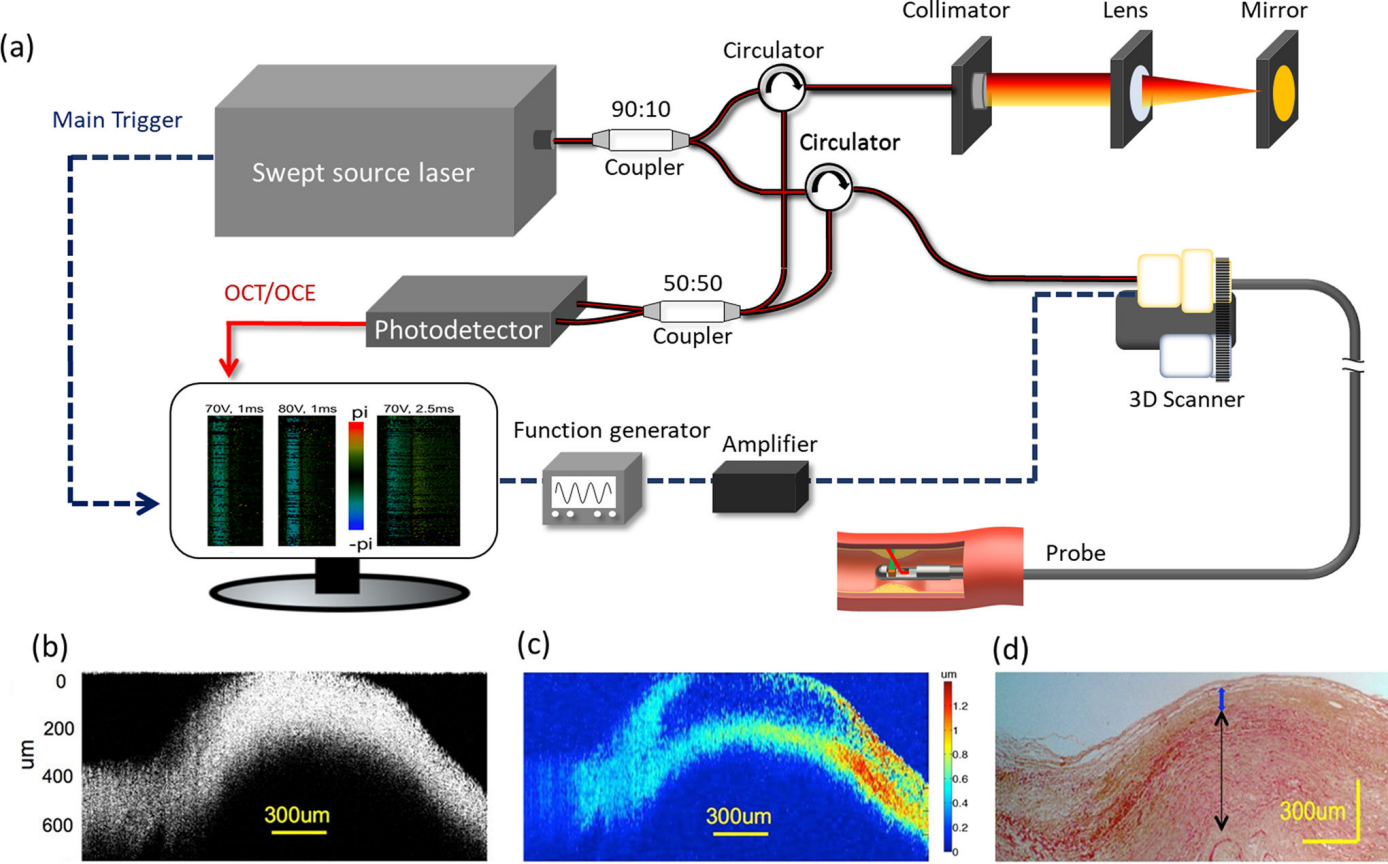
Integrated IVOCT and IVOCE imaging system and results from a cadaver coronary artery. (a) System schematic. (b) IVOCT image. The reduced displacement on the left side of the image indicates a plaque formation. (c) IVOCE image.(d) Histology. Adapted from Ref. 16.

To explore the mechanical properties of coronary arteries, IVOCT-OCE has been proposed.^16,31^ In addition to visualization of tissue morphology provided by OCT, OCE enables spatial mapping of arterial wall elasticity. Because the stability of plaque depends on tissue biomechanical properties, imaging and characterization of biomechanical properties provide a direct assessment of plaque vulnerability. A schematic of an IVOCT-OCE system setup is depicted in Fig. 3(a) in which a swept-source OCT system is used for both OCT and OCE imaging. Additionally, a spectrometer-based OCT system can be used instead of a swept-source laser to provide higher phase stability. Typically, acoustic radiation force (ARF) excitation is used to generate localized displacement for tissue elasticity calculations. Alternatively, one can also use other forces, such as pressure change generated by flushing or intrinsic cardio function, for OCE excitation. The trigger signal from the swept-source laser is used to synchronize a function generator to create ARF and for data acquisition. Different scanning protocols (such as M-B mode) can be used to extract phase-resolved Doppler signals for calculating Young’s modulus.

Figures 3(b)–3(c) show the representative images of the coronary artery from a human cadaver obtained using an IVOCT-OCE system. From the OCT image alone [Fig. 3(b)], it is difficult to identify the plaque. However, OCE [Fig. 3(c)] demonstrates a reduced displacement on the left side of the image, suggestive of a plaque formation. In addition, OCE also reveals a distinct layer with small displacement within the middle bulging section, indicating the possible presence of a fibrous plaque layer. The corresponding histology shows the intimal thickening and atheroma, which is consistent with the IVOCE image. This study demonstrates that integration of IVOCT and OCE has the potential to identify lipid content as well as to visualize tissue morphology. To date, only *ex vivo* imaging has been achieved for feasibility verification. For clinical translation, several improvements are necessary. Firstly, the current imaging speed (8 minutes per frame in the reported study) is insufficient for real-time imaging. Incorporation of a high-speed laser and a high efficiency US transducer may enable a B-M mode scanning protocol in which a 2D elasticity map can be obtained with a single shot of ARF excitation. Additionally, high-speed imaging can also reduce the interference from environmental vibration. Secondly, the diameter of the probe needs to be minimized to ~ 1 mm in order to be compatible with a clinical catheter. Thirdly, the penetration depth needs to be improved for accurate assessment of plaque biomechanical properties. The currently reported IVOCT-OCE system uses a spectrometer-based OCT system centered at an 890 nm wavelength, which limits the penetration depth. Therefore, using a longer center wavelength light source (such as a 1.7 *μ*m swept-source laser) in IVOCT-OCE could be the future direction to extend the penetration depth. Finally, the phase stability can be further improved in order to provide more precise measurements of elasticity; a micromotor and a common-path configuration could be a possible solution to enhance the phase stability.

### (4) IVPA-US

**Fig. 4.**
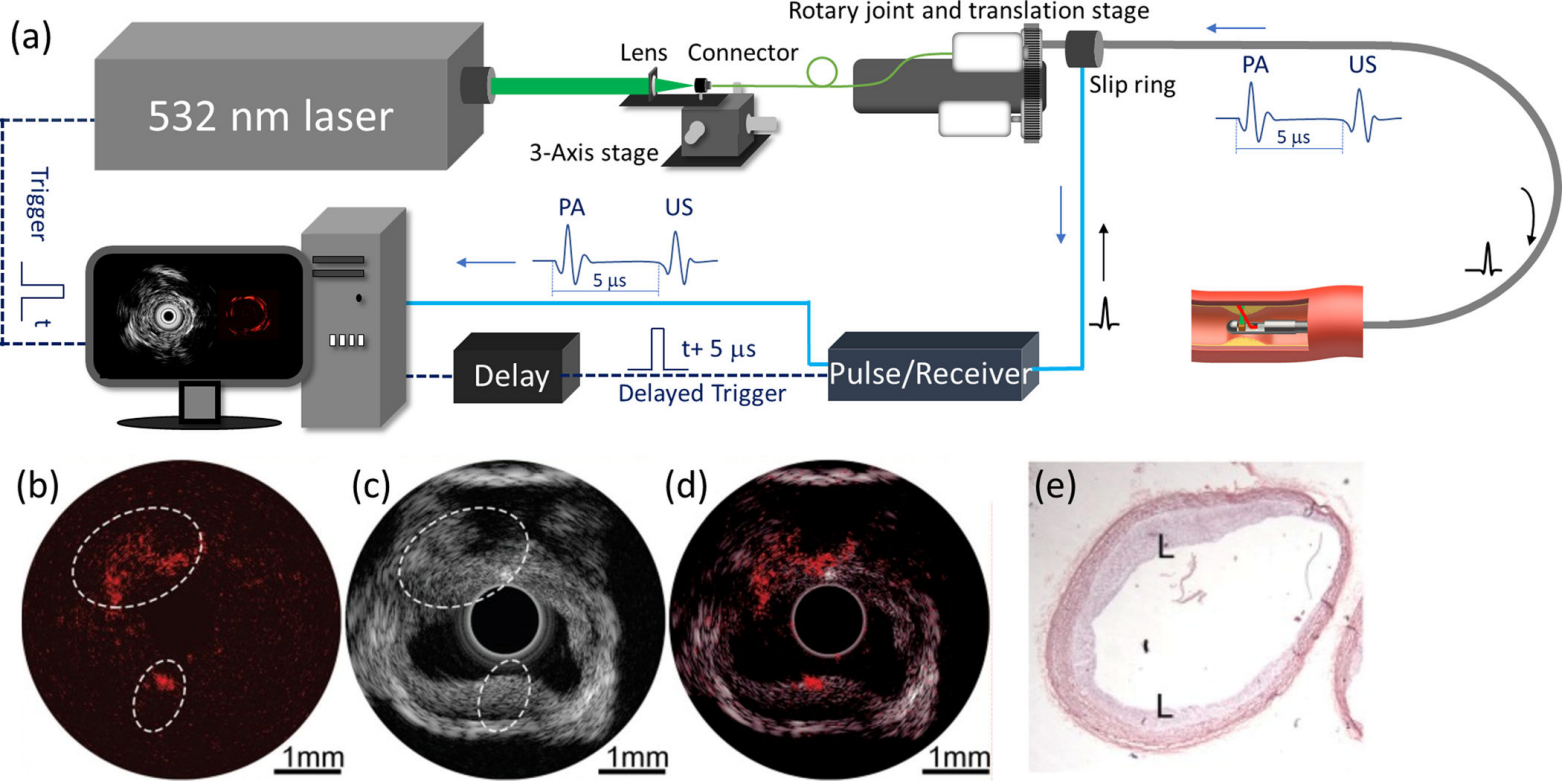
Integrated IVPA-US of an atherosclerotic rabbit abdominal aorta. (a) System schematic. (b) PA image. (c) US image. (d) Histology. L: lipid deposition. High-intensity PA signals (white dashed circles) are observed, and intimal thickening can be found in the corresponding locations in the US image as well as confirmed by histology. Adapted from Ref. 17.

IVPA imaging uses optical absorption as a contrast and can characterize the plaque by mapping the depth-resolved composition. As PA imaging requires the use of US transducers, US imaging can be incorporated seamlessly. IVPA-US can provide tissue composition and morphology simultaneously.^17,28,50–56^ Figure 4 depicts the schematic of a typical IVPA-US system setup.^57^ A nanosecond pulsed laser is used to provide PA signal excitation at the arterial wall. Since IVPA and IVUS imaging share the same US transducer, a delay unit is often used to delay the main trigger signal from the laser to synchronize the US pulse emission in order to separate the generated PA and US signals. Different excitation wavelengths can be selected for different tissue targets, such as intimal and lipid. Currently, 1.2 *μ*m and 1.7 *μ*m lasers are often used for mapping lipids in an atherosclerotic plaque as these two wavelengths are at the peak of the lipid absorption spectrum. In addition, multiple wavelengths can be used for spectroscopic IVPA imaging to further differentiate the lipid components. Figures 4(b)–4(e) show the representative PA, US, combined, and histology images of an atherosclerotic rabbit abdominal aorta. In the PA image [Fig. 4(b)], high-intensity PA signals are observed, denoted by the white circles, where the intimal thickening can be found in the corresponding locations in the US image, suggesting the presence of lipid plaque. Histology confirms the lipid deposits at the same locations.

Although mapping lipid has been demonstrated by *ex vivo* and *in vivo* atherosclerotic rabbit studies, moving forward to larger animal studies and clinical trials still require further improvements. Chiefly, high-speed imaging is the main challenge for IVPA as there is a trade-off between imaging speed and sensitivity. Currently, the highest *in vivo* imaging speed reported is 20 fps but only with a 250 A-line per frame.^58^ To achieve high speed imaging, heavy water (deuterium oxide) was used to enhance the sensitivity due to the low excitation pulse energy which is not ideal due to the potential risks and expensive costs associated with heavy water.^56,58^ A high-repetition-rate with a high-pulse-energy nanosecond laser at an optimal wavelength for lipid contrast could be a potential solution. Additionally, visualizing depth-resolved tissue composition with high resolution is necessary for accurate quantification of lipid components. In order to map the entire plaque, a probe design with a broad illumination scheme (i.e., not optically focused) is often utilized which causes a reduced lateral resolution. A hybrid optical and acoustic resolution endoscope with an improved penetration depth while maintaining the resolution has been developed to visualize the vasculature of a mouse ear,^59^ which may be adapted for intravascular imaging to overcome the current limitations of IVPA imaging.

### (5) Other IVUS-Based Dual-Modality Systems

Several other dual-modality intravascular imaging systems have been proposed to meet different clinical needs. These systems, including IVUS-NIRF, IVUS-NIRS, IVUS-FLIM (fluorescence-lifetime imaging microscopy), are able to visualize the entire structure and composition of coronary artery simultaneously.^24,60–63^ Among them, IVUS-NIRS has been commercialized and approved for clinical use in the United States. Similar to IVOCT-NIRF, the lack of depth-resolved molecular contrast limits their accuracy, and the penetration depth of the optical imaging may not be sufficient to cover the entire region of interest, especially for FLIM. In addition, the measurement of fibrous cap thickness and the evaluation of stent implantation cannot be achieved due to the low resolution of IVUS.

### (6) Tri-Modality Techniques

**Fig. 5.**
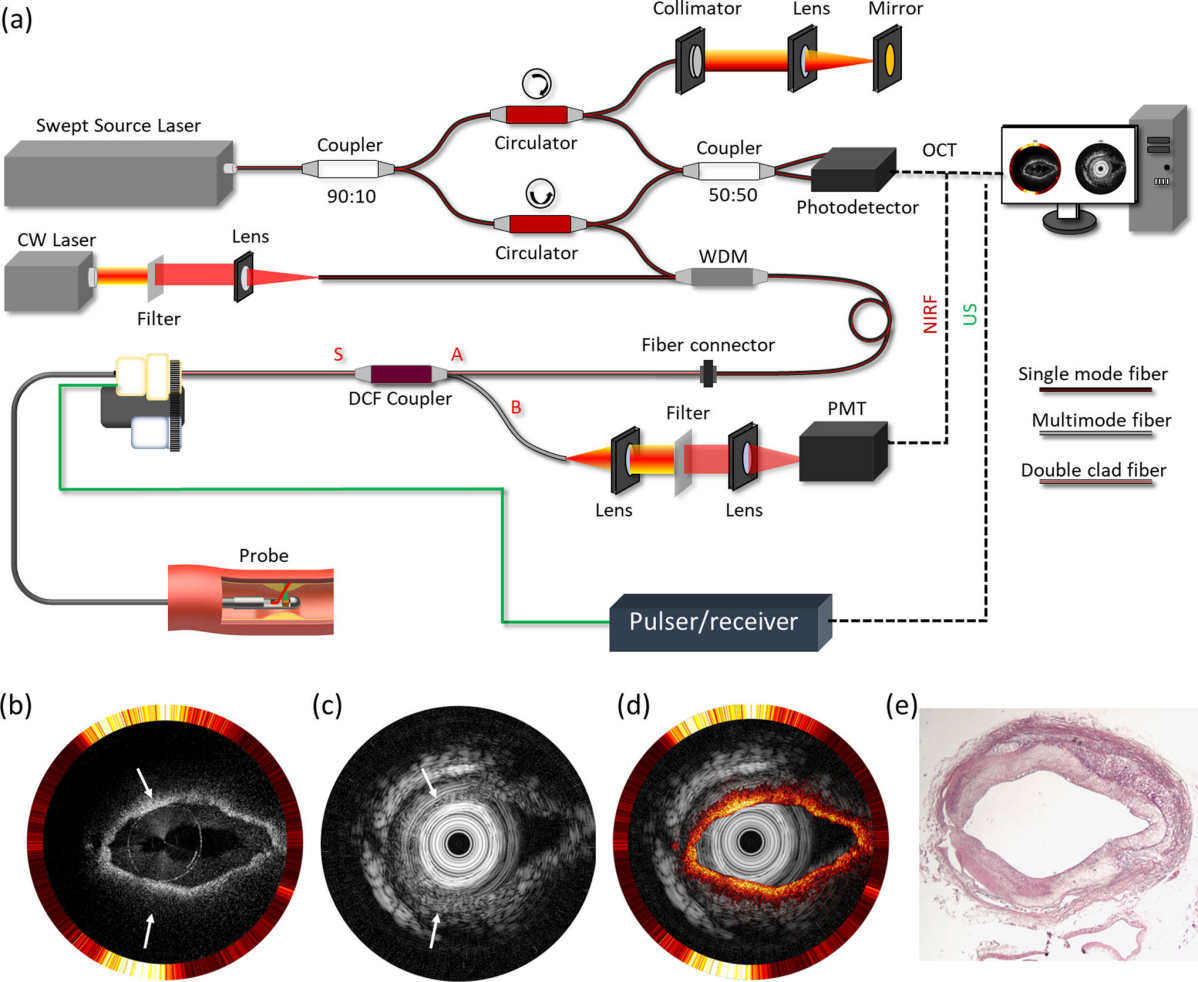
Integrated IVOCT-US-NIRF imaging of an ex vivo rabbit aorta. (a) System schematic. (b) IVOCT-NIRF (inner: IVOCT, outer: NIRF). (c) US image. (d) Overlaid image of (b) and (c). (e) Histology. WDM: wavelength-division multiplexing. DCF: double clad fiber. PMT: photomultiplier tube. CW: continuous wavelength. OCT: optical coherence tomography. US: ultrasound. NIRF: near infrared fluorescence.

Several tri-modality intravascular imaging systems have been reported.^9,64,65^ Figure 5(a) shows the schematic of a tri-modality IVOCT-US-NIRF system. The trigger signal from a swept-source laser is used to synchronize the US pulse emission and data acquisition. Similar to IVOCT-NIRF, a WDM and DCF coupler are used to combine, propagate, and separate the OCT and NIRF signals. OCT, US, and NIRF each have the capability to detect thin fibrous caps, large lipid pools, and inflammation, respectively. Figures 5(b)–5(e) are the imaging results from an atherosclerotic rabbit aorta with this tri-modality system. In IVUS and OCT images, the micro (i.e., fibrous caps) and gross (i.e., intimal thickening) structures of plaque can be visualized. In NIRF, the sites with inflammation are identified.

**Fig. 6.**
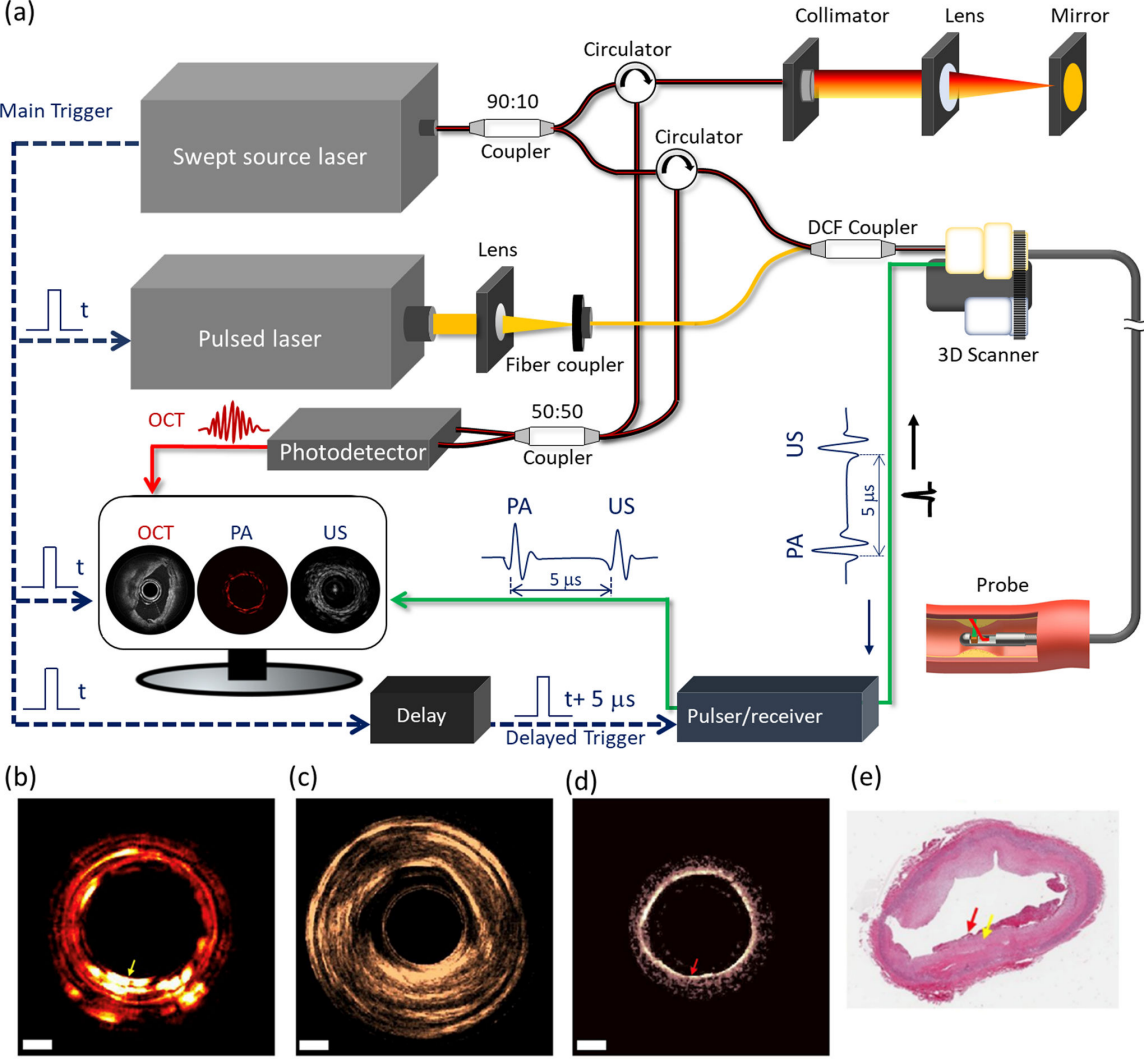
Integrated IVOCT-PA-US of human artery. (a) System schematic. DCF: double-clad fiber. (b) PA image. (c) US image.(d) OCT image. (e) Histology. Scale bars: 2 mm. Adapted from Ref. 65.

Although IVOCT-US-NIRF can provide molecular contrast, the composition map does not contain depth information which limits the accuracy. In addition, NIRF requires a contrast agent, which increases the procedure risk. Hence, tri-modality IVOCT-PA-US has been proposed to address these issues as it provides depth-resolved molecular contrast and morphology of coronary arteries without the use of a contrast agent.^65^ Figure 6 shows a typical IVOCT-PA-US system design.^66^ The trigger signal from a swept-source laser is used to synchronize the nanosecond pulsed laser and delay unit for US pulse generation as well as data acquisition. For complete integration, a DCF coupler is used to couple the OCT and the PA light into the core and inner cladding of the DCF, respectively. To achieve optical-resolution PA imaging, a WDM (instead of a DCF coupler) can be used to combine two laser beams into a single mode fiber.

Dai et al. presented the *ex vivo* experimental results [Figs. 6(b)–6(e)] obtained using the IVOCT-PA-US system and demonstrated its capability for atherosclerosis diagnostics.^65^ Nonetheless, an improved imaging speed is necessary to facilitate clinical translation, and an imaging probe with a reduced form factor is also needed to be compatible with clinical catheters. Lastly, further verification and performance quantification through *in vivo* animal studies are also required.

## 3. Imaging Probe Designs

An optimal probe design is essential for high-quality images, which is perhaps the most challenging part in multimodal intravascular imaging. Typically, an optical and/or an acoustic sensor is mounted at the distal tip of the imaging probe. In order to access the small branches of coronary arteries, the imaging probe needs to satisfy an outer diameter (OD) of < 1 mm and a rigid length of < 5 mm.^18,52^ These dimensional limits bring further challenges to optimal imaging quality, such as lateral resolution and detection efficiency.

Many multimodal imaging probes have been proposed, each with its own advantages and limitations, which are summarized in Table 2. These designs can be categorized into two groups based on component alignment: sequential and coaxial. In coaxial alignment, optical and acoustic beams share the same path, as shown in Table 2(1)–(4). This configuration allows for automatic image co-registration and high detection efficiency. In the case of PA and OCE, coaxial alignment can maximize the imaging range. For the design shown in Table 2(4), the acoustic probe can also be placed under the optical probe. Additionally, a dual-element acoustic probe can be incorporated instead of a single acoustic transducer to improve sensitivity.^67^ In sequential alignment, the optical sub-probe and acoustic sub-probe are arranged in series, as shown in Table 2(5)–(8). For the designs depicted in Table 2(5)–(7), either the optical beam and/or the acoustic beam is tilted at a small angle to achieve an overlap between the two beams at the designed working distance for image co-registration (and high-efficiency detection for PA). However, this arrangement creates a longitudinal offset between the two beams, limiting the imaging range of PA and OCE. Furthermore, this configuration requires additional steps of offsetting for image co-registration. For the design shown in Table 2(8), the optical and the US component are oppositely oriented (i.e., back-to-back) to eliminate the longitudinal offset between the two beams; however, this design is not applicable for PA and OCE imaging, which requires beam overlap.

The optical sub-probe can also be divided into two groups based on illumination scheme: broad illumination and focusing illumination. The broad illumination scheme, shown in Table 2(5) and (9), has a low lateral resolution but can achieve a small form factor with a minimal OD and rigid length. In the focusing illumination scheme, the light focus can be realized by using a gradient lens, gradient fiber, or a ball lens; this scheme has an improved lateral resolution although at the cost of an increased OD and rigid length. Additionally, the numeric aperture (NA) of the gradient fiber and ball lens determines the lateral resolution so the probe size has to be increased when a high lateral resolution is required.

Two commonly used scanning methods for these designs include proximal and distal scanning. In a proximal scanning setup, an optical and electrical rotary joint is used to rotate the entire imaging probe to perform a radial scan.^8^ To achieve an efficient torque transmission from the rotary joint to the probe tip, a double-wrap torque coil is used to enclose the fiber and cable. While this scanning method can allow the imaging probe to have a small form factor (including OD and rigid length), nonuniform rotation distortion (NURD) is a concern due to the tortuous cardiovascular vessels. In the distal scanning scheme, a micromotor is assembled at the tip of the imaging probe to drive the mirror for radial scanning.^68^ While this can achieve high-speed imaging with uniform rotation, the rigid length is longer due to the micromotor. Moreover, a waterproof micromotor is required for an IVUS-based imaging probe, which increases the cost.

## 4. Discussion

Multimodal technology overcomes the limitations of individual intravascular imaging modality, providing more comprehensive information on plaque morphology and/or composition for better characterization of vulnerable plaques. Various combinations of different modalities have been proposed and developed to aim for advancing the clinical management of atherosclerosis. For instance, IVUS-NIRS and IVUS-OCT have been commercialized and are currently undergoing clinical trials.

Morphology, composition, and mechanical properties of coronary arteries are the key determining factors of vulnerable plaques. Morphological information, including the microstructures, can aid in determining the key physical landmarks of the disease. Compositional information allows for the identification of lipid components and macrophage and, therefore, plays an important role in the evaluation of plaque vulnerability. IVUS-OCT can image coronary arteries at both the acoustic and optical scales to obtain both gross and micro structures of the arterial wall, and compositional information can also be inferred based on the structural data, although with a limited accuracy.

IVUS-NIRF/NIRS and IVOCT-NIRF/NIRS have the capability of visualizing both morphology and composition of the plaque directly, but they lack the ability to simultaneously visualize both the micro and the gross structures. To address this limitation, tri-modality IVUS-OCT-NIRF was proposed in 2014 to directly detect the main determining factors related to atherosclerosis. However, NIRF/NIRS does not yield depth information, and currently it remains unclear if near-infrared light can provide adequate penetration for imaging lipid in deeper tissue. PA imaging overcomes the limitation of NIRF/NIRS as it is able to map depth-resolved composition of the plaque. Therefore, IVPA-US allows for visualization of both morphology and depth-resolved composition. Additionally, IVOCT has also been integrated into IVPA-US to provide microstructural information, enhancing the capability of plaque characterization. Furthermore, the association between mechanical properties and plaque vulnerability have been demonstrated, and IVOCE, first reported in 2017, has been applied in intravascular imaging to quantify the elasticity of plaque.

An ideal intravascular imaging technique should have high resolution, large penetration depth, depth-resolved molecular contrast, and elasticity quantification. A combined IVPA-US-OCT-OCE system, if achieved, can provide a means to acquire these attributes. In such a system, an US transducer can be used to detect the PA signals and perform US imaging as well as provide ARF excitation for OCE. A DCF fiber or a single mode fiber can be used to propagate OCT and PA light simultaneously. A properly designed imaging probe is the key component for realizing multimodal imaging systems, and several requirements must be met in order to facilitate clinical translation. Firstly, a small form factor must be achieved (i.e., < 1 mm OD and < 5 mm rigid length) to ensure that the probe can be advanced through the artery smoothly. Secondly, accurate co-registration images are essential. A coaxial probe design allows for an optimal overlap between the acoustic beam and optical beam, but the acoustic attenuation caused by multi-reflection from the mirror and side lobe effect from the ring US transducer can affect the imaging quality. Finally, high-speed imaging is required in order to reduce the procedure risks and enhance measurement accuracy by reducing the influence from environmental vibration. A waterproof and short rigid length micromotor may be the solution for enabling high-speed and stable scanning as well as eliminating NURD.

## 5. Conclusions

Intravascular multimodal imaging technology has great potential for improving disease diagnostics and clinical management of atherosclerosis. Several animal studies and clinical trials have demonstrated its efficacy in characterizing atherosclerotic plaques with improved accuracy. Further improvements are required to overcome the current limitations of the multimodal system and probe designs. Finally, a quad-modality system, such as IVPA-US-OCTOCE, may be a promising technique for providing comprehensive plaque evaluation.

## Figures and Tables

**Table 1.** Comparison of different intravascular imaging technologies.

	Identification of vulnerable plaque					Evaluation of stent implantation
	Thin fibrous cap	Large lipid pool		Inflammatory reaction	Mechanical property	
		Composition	Dimension			
IVOCT	**	*		*		**
IVUS		*	**			*
NIRF		*		**		
NIRS (FLIM)		**				
IVPA		**	* *			*
IVOCE	**	*			**	

Notes:

** indicates excellent performance;

* moderate performance.

**Table 2.** Summary of intravascular imaging probes.